# Expression of Myeloid Antigen in Neoplastic Plasma Cells Is Related to Adverse Prognosis in Patients with Multiple Myeloma

**DOI:** 10.1155/2014/893243

**Published:** 2014-06-04

**Authors:** Hyoeun Shim, Joo Hee Ha, Hyewon Lee, Ji Yeon Sohn, Hyun Ju Kim, Hyeon-Seok Eom, Sun-Young Kong

**Affiliations:** ^1^Department of Laboratory Medicine, Center for Diagnostic Oncology, Research Institute and Hospital, National Cancer Center, 323 Ilsan-ro, Ilsandong-gu, Goyang, Gyeonggi-do 410-769, Republic of Korea; ^2^Hematologic-Oncology Clinic, Center for Specific Organs Cancer, Research Institute and Hospital, National Cancer Center, 323 Ilsan-ro, Ilsandong-gu, Goyang, Gyeonggi-do 410-769, Republic of Korea; ^3^Hematologic Malignancy Branch, Research Institute and Hospital, National Cancer Center, 323 Ilsan-ro, Ilsandong-gu, Goyang-si, Gyeonggi-do 410-769, Republic of Korea; ^4^Translational Epidemiology Research Branch, Research Institute and Hospital, National Cancer Center, 323 Ilsan-ro, Ilsandong-gu, Goyang-si, Gyeonggi-do 410-769, Republic of Korea

## Abstract

We evaluated the association between the expression of myeloid antigens on neoplastic plasma cells and patient prognosis. The expression status of CD13, CD19, CD20, CD33, CD38, CD56, and CD117 was analyzed on myeloma cells from 55 newly diagnosed patients, including 36 men (65%), of median age 61 years (range: 38–78). Analyzed clinical characteristics and laboratory parameters were as follows: serum **β**2-microglobulin, lactate dehydrogenase, calcium, albumin, hemoglobin, serum creatinine concentrations, bone marrow histology, and cytogenetic findings. CD13+ and CD33+ were detected in 53% and 18%, respectively. Serum calcium (*P* = 0.049) and LDH (*P* = 0.018) concentrations were significantly higher and morphologic subtype of immature or plasmablastic was more frequent in CD33+ than in CD33− patients (*P* = 0.022). CD33 and CD13 expression demonstrate a potential prognostic impact and were associated with lower overall survival (OS; *P* = 0.001 and *P* = 0.025) in Kaplan-Meier analysis. Multivariate analysis showed that CD33 was independently prognostic of shorter progression free survival (PFS; *P* = 0.037) and OS (*P* = 0.001) with correction of clinical prognostic factors. This study showed that CD13 and CD33 expression associated with poor prognosis in patients with MM implicating the need of analysis of these markers in MM diagnosis.

## 1. Introduction

Flow cytometry (FCM) is widely used for the diagnosis and monitoring of hematological disorders, such as acute leukemias or lymphomas, in order to detect and characterize abnormal compartments or to enumerate rare events [1]. Flow cytometric analysis of neoplastic plasma cells in patients diagnosed with multiple myeloma (MM) can distinguish clonal cell populations and can be used to determine the numbers of neoplastic cells and to monitor residual disease during treatment [2].

In plasma cells, aberrant expression of CD56 and CD28 but lack of CD19 and CD27 showed the association with malignancy [3]. Downregulation of CD56 and a higher expression of CD44 have been associated with extramedullary spreading of malignant plasma cells [4, 5] and expression of CD28 has been related to disease activity [6, 7]. Though many studies have reported the associations between the expression of several antigens, including CD19, CD28, CD56, and CD117, and patient prognosis [8–10], no consensus has been reached regarding the expression status of antigens and their clinical relevance. Here we evaluated the impact of antigen expression of neoplastic plasma cells on survival of patients diagnosed with MM.

## 2. Materials and Methods

Bone marrow (BM) aspiration samples were obtained from 55 patients newly diagnosed with MM from November 2007 to March 2013. Flow cytometric analyses perfomed in condition of plasma cells over 5% in the specimens. Whole erythrocyte-lysed BM samples were stained using the following four-color combinations of antibodies (FITC/PE/PerCP/APC): CD19/CD117/CD138/CD45, CD20/CD33/CD138/CD45, CD38/CD13/CD138/CD45, -/CD56/CD138/CD45, and cyto-Kappa/cyto-Lambda/CD138/CD45. Antibody combinations were changed once from anti-CD38/CD13/CD138/CD45 to anti-CD38/CD28/CD138/CD45 during the study period. To assess antigens expression an aliquot of approximately 1 × 10^6^ cells was labeled with preconjugated monoclonal antibodies in accordance with the manufacturer's recommendations (BD Biosciences, USA). The cells were then washed with phosphate buffered saline (PBS). For CD138 gating, at least 1 × 10^3^ events per tube were acquired. Analyses were carried out using the FACS Diva software (BD Biosciences). Cells were also incubated with irrelevant isotype-matched antibodies to determine background fluorescence. Side scatter and high level expression of CD138 were used to gate each preparation of plasma cells. CD138 gated cells from patients with MM were retrospectively defined as neoplastic plasma cells when it was diagnosed as monoclonal gammopathy on serum and/or urine electrophoresis and light chain restriction on immunohistochemical staining of BM biopsy section. Positivity for antigen expression on flow cytometry was defined as staining of >20% of the cells.

Patient characteristics were retrospectively evaluated, including laboratory parameters including serum *β*2-microglobulin, calcium, albumin, hemoglobin, lactate dehydrogenase (LDH), serum creatinine concentrations, and immunoglobulin type of monoclonal protein. Fifty-five patients with MM were analyzed, 36 males (65%) and 19 females (35%), of median age 61 years (range: 38–78 years) (Table 1). BM histologic findings were classified as mature (*n* = 39), immature (*n* = 9), plasmablastic (*n* = 2), or pleomorphic (*n* = 5) myeloma cell types. Infiltration was categorized by interstitial (*n* = 16), focal (*n* = 3), or diffuse (*n* = 36) pattern. The FISH panels included* p53* (17p13),* Rb1* (13q14),* IGH/FGFR* t(4;14), and trisomy 1q (1q21). Cytogenetic abnormalities of t(4;14) or del(17p) were designated as high risk [11].

The initial treatment regimen consisted of including thalidomide and dexamethasone (57%), bortezomib (19%), combination of thalidomide and bortezomib (6%), lenalidomide (4%), and others (14%). Autologous peripheral blood stem cell transplantation (PBSCT) was performed in 33% of patients. Stage was classified by the international staging system and Durie-Salmon staging system [12, 13]. Risk group and disease progression were defined according to the International Myeloma Working Group (IMWG) risk stratification and response criteria for MM, respectively [14, 15].

Progression-free survival (PFS) was calculated from the date of diagnosis to the date of relapse, disease progression, or death from any cause. Overall survival (OS) was calculated as the time from the date of diagnosis to death from any cause. PFS and OS were determined by the Kaplan-Meier method and log-rank test. Continuous variables were compared using independent *t*-tests or Mann-Whitney tests and categorical variables using Pearson chi-square or Fisher's exact tests. Multivariate analysis was performed using Cox regression analysis. Data were analyzed using SPSS 21 software (IBM Corp. 2012, IBM* SPSS Statistics, version 21.0,* Armonk, NY). This study was approved by the institutional review board of National Cancer Center of Korea (NCCNCS-13-774).

## 3. Results

**Figure 1:**
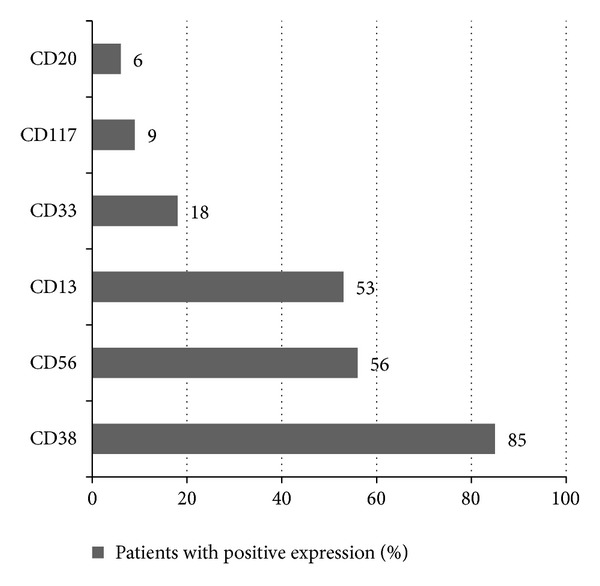
Frequency of antigen expression in patients newly diagnosed with multiple myeloma. CD56 and CD13 were the most common aberrant antigens in neoplastic plasma cells (56% and 53%, resp.), followed by CD33, CD117, and CD20. CD13 and CD33, the traditional myeloid markers, showed relatively high prevalence.

The expression of CD38 was detected in 85% of cases (47 of 55) in CD138+ gated plasma cells. The expression of CD56, a marker involved in anchoring plasma cells to stromal structures, was found in 56% of cases (31 of 55). CD13 and CD33, the markers of myeloid lineage, were detected in 53% (20 of 38) and 18% (10 of 55) of cases, respectively. CD117, a tyrosine kinase receptor was detected in 9% (5 of 54). CD20, an antigen associated with the early stages of B-cell maturation, was detected in only 6% (3 of 55) of cases (Figure 1).

CD33 positivity was significantly associated with higher serum calcium (*P* = 0.049) and LDH (*P* = 0.018) concentrations (Table 2). Moreover, immature and plasmablastic cell type was more frequently observed in CD33+ than CD33− patients (*P* = 0.022). CD13 expression did not show the association with clinical characteristics except infiltration pattern (*P* = 0.046). High risk cytogenetics, IMWG risk stratification, ISS stage, or Durie-Salmon stage has no significant difference in expression of myeloid antigens. Univariate analysis showed that CD13 positivity (*P* = 0.008), *β*2-microglobulin > 3.5 mg/dL (*P* = 0.003), and LDH > 202 U/L (*P* = 0.007) were significantly associated with shorter PFS. In addition, CD13 positivity (*P* = 0.025), CD33 positivity (*P* = 0.001), *β*2-microglobulin > 3.5 mg/dL (*P* = 0.007), and LDH > 202 U/L (*P* < 0.001) were significantly associated with shorter OS.

The prognostic indicators found to be significant in univariate analyses were included in multivariate analyses. CD33 positivity was the factor independently prognostic for OS (HR: 14.2, 95% CI: 3.3–61.8, *P* < 0.001). *β*2-Microglobulin > 3.5 mg/dL was another independent prognostic factor associated with PFS (HR: 6.93, 95% CI: 2.0–24.1, *P* = 0.002) (Table 3).

**Figure 2:**
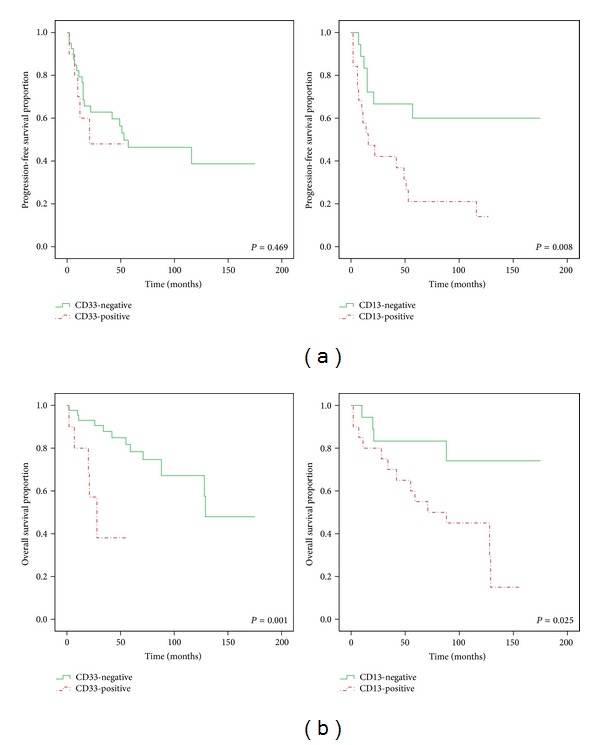
Kaplan-Meier analysis of (a) progression free survival (PFS) and (b) overall survival (OS) in groups of patients positive and negative for CD33 and CD13. CD33 expression demonstrates a potential prognostic impact and was associated with lower OS (*P* = 0.001). Patients with CD13 associated with significantly shorter PFS times (*P* = 0.008), not only lower OS (*P* = 0.025).

CD33 and CD13 expression were associated with lower OS (*P* = 0.001 and *P* = 0.025) at a median followup of 51 months. The estimated 2-year OS rate was significantly lower in CD33+ than in CD33− patients (38% versus 78%, *P* = 0.046) and CD13+ than in CD13− patients (55% versus 83%, *P* = 0.046). PFS was significantly shorter in CD13+ than CD13− patients (*P* = 0.008, Figure 2). Other antigens did not influence OS or PFS as follows: CD56 (*P* = 0.252, *P* = 0.417), CD117 (*P* = 0.912, *P* = 0.975), and CD20 (*P* = 0.679, *P* = 0.253).

**Figure 3:**
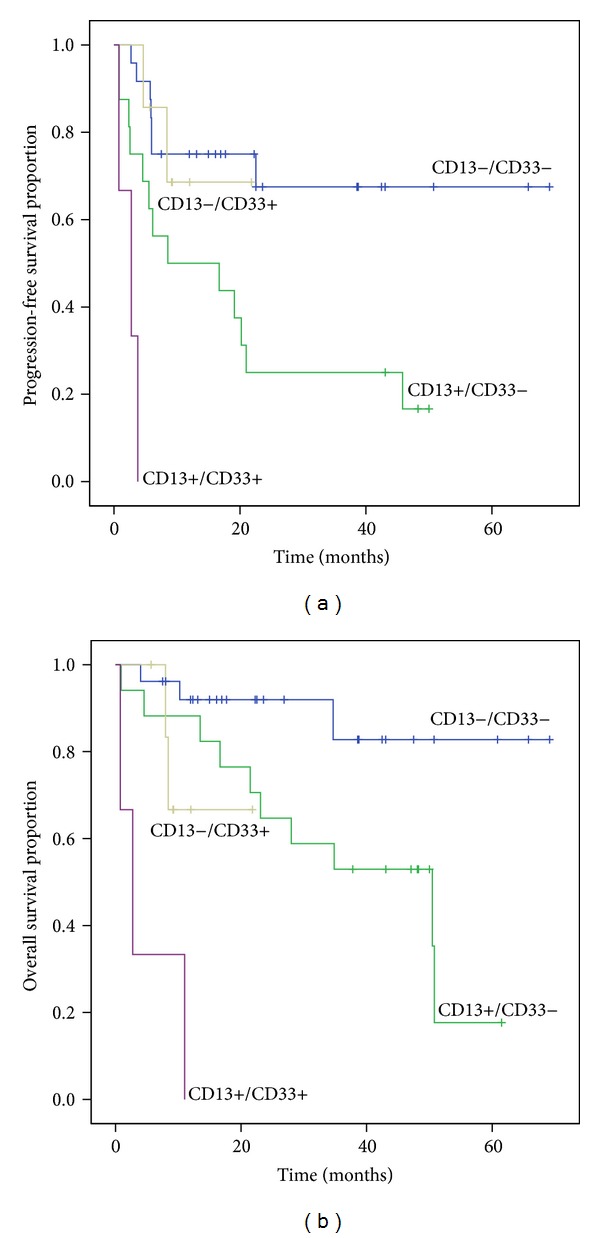
Kaplan-Meier analysis of (a) PFS and (b) OS in groups of patients with CD13−/CD33−, CD13−/CD33+, CD13+/CD33−, and CD13+/CD33+. The CD13+/CD33+ group showed significantly shorter PFS and OS than other groups: CD13−/CD33− group (*P* < 0.001 in PFS and OS), CD13+/CD33− group (*P* = 0.013 in PFS and *P* < 0.001 in OS), and CD13−/CD33+ group (*P* = 0.001 in PFS, *P* = 0.049 in OS). CD13+/CD33− group showed significantly shorter PFS and OS than CD13−/CD33− group (*P* = 0.006 in PFS, *P* = 0.020 in OS).

The numbers of patients with CD13+/CD33+, CD13+/CD33−, CD13−/CD33+, and CD13−/CD33− groups were 3, 17, 3, and 15, respectively, and the CD13+/CD33+ group showed significantly shorter PFS and OS than other groups (Figure 3).

## 4. Discussion

This study showed myeloid antigens CD13 and CD33 were associated with poor prognosis in MM patients. Univariate analysis showed that both antigens were associated with short OS; moreover multivariate analysis showed that CD33 expression was independent prognostic factor for poor prognosis. Both CD13+/CD33+ group showed significantly short OS and PFS and it suggests that expression of CD13 and CD33 has additive effect on unfavorable prognosis even though each group was not big enough to conclude. Though CD33 expression on plasma cells showed significant difference in OS, it did not show correlation with PFS. Since our study has limitation which included several treatment regimens, PFS which reflects more treatment response rather than biologic entity of myeloma did not reached the significant level.

With correlation of clinical parameters, the previous study has shown CD33 positivity was associated with higher serum LDH and *β*2-microglobulin concentrations and higher incidence rates of anemia or thrombocytopenia [16], and this study showed a significant association between CD33 positivity and higher serum LDH concentration (*P* = 0.018). For cytogenetic risk, there was the study showing higher incidence of t(4;14) in CD33-positive patients [17]; however, the association with t(4;14) was not observed in our study.

For mechanism of CD13 and CD33 in myeloma cells, there was no suggested pathway. The normal function of CD13 and CD33 in myeloid lineage is a zinc-dependent metalloproteinase anchored to cells as a type II transmembrane protein [18] and a sialic acid dependent cell adhesion molecule with a cytoplasmic tail bearing two tyrosine residues [19] which recruits Src homology-2 domain-containing tyrosine phosphatases [20]. These markers have been shown correlation with cancer in increased motility of lung cancer cells resulting in high invasiveness [21] and drug resistance and refractoriness with significantly lower 1-year survival rate in MM [16].

The clue why our study represented correlation with prognosis lied in plasma cell type and infiltration pattern. Morphologic subtype of MM plasma cells and infiltration pattern were reported as prognostic factors by the previous studies, which showed plasmablastic cells and diffuse infiltrations were associated with poor prognosis [22–24]. In the present study, immature and plasmablastic types of plasma cells were significantly associated with CD33 positivity. This implicated CD33+ myeloma associated with poorly differentiated neoplastic plasma cell type. Also CD13+ myeloma patients showed either focal or diffuse pattern of infiltration which suggests the association of antigen expression with infiltration characteristics.

For other antigen expressions, we found that 56% of patients were positive for CD56, 53% for CD13, 18% for CD33, 9% for CD117, and 6% for CD20. In comparison, previous studies have found that 60–75% of MM patients were positive for CD56, 18–35% for CD33, 32% for CD117, and 17–30% for CD20 [9, 17, 25–27]. These discrepancies in the antigen expression frequencies could result from the differences in the definition of neoplastic plasma cell; some studies exclude CD138+, CD19+, CD45+, CD27+, CD56−, and CD20− cells because they were regarded as normal plasma cells [8], but we included all CD138+ gated cells. The immunophenotypic definition of neoplastic plasma cells remains still unclear, because antigen expression profiles in normal or benign plasma cells are not uniform. Other traditional myeloid markers have shown divergent impact in patients with MM. CD117, c-kit receptor, has been associated with good prognosis [3, 28] or not associated with prognosis [29–31]. The mechanism was explained as follows: CD117 expression might act as anchor molecule resulting in a decrease spread of plasma cells for good prognosis [28]. In this study, CD117+ patients did not display neither different disease characteristics nor a worse outcome. It might be due to low frequency of CD117 positivity in the present study, which could result from different destination of neoplastic plasma cells.

The major limitation of this study was the lack of homogenous treatment. However, CD33 expression was associated with significant short OS in both patients who underwent PBSCT (*n* = 16, *P* < 0.001) or who did not (*P* = 0.046). Thus, our findings implicate the need of analysis of these markers in MM diagnosis.

## 5. Conclusion

In conclusion, this study showed that the expression of CD13 and CD33 in neoplastic plasma cells from patients with MM was associated with poor prognosis independently of other prognostic factors. Further study is needed to clarify the role of these markers in MM pathogenesis.

## Figures and Tables

**Table 1:** Clinical characteristics of the 55 patients with multiple myeloma.

Characteristics	Number (%) or median (range)
Number of patients	55
Age	61 (38–78)
Gender (male : female)	36 : 19 (65 : 35)
Durie-Salmon stage (I : II : III)	5 : 10 : 40 (9 : 18 : 73)
ISS stage (I : II : III)	22 : 18 : 15 (40 : 33 : 27)
Calcium (mg/dL)	9.1 (7.2–13.0)
Creatinine (mg/dL)	1.2 (0.7–3.9)
Albumin (mg/dL)	4.0 (2.3–4.9)
*β*2-Microglobulin (mg/dL)	3.8 (1.6–19.0)
Hemoglobin (g/dL)	10.3 (6.0–16.2)
Lactate dehydrogenase (U/L)	167 (79–1832)
C-reactive protein (mg/dL)	0.27 (0–10.01)
IgG : IgA : IgM : IgD : IgE : light* : biclonal	33 : 11 : 0 : 0 : 0 : 9 : 2 (60 : 20 : 0 : 0 : 0 : 16 : 4)
Kappa : Lambda (electrophoresis)	23 : 22^†^ (51 : 49)
Plasma cell type	
Mature	39 (71)
Immature	9 (16)
Plasmablastic	2 (4)
Pleomorphic	5 (9)
Infiltration pattern	
Interstitial	16 (29)
Focal	3 (5)
Diffuse	36 (66)
Frequency of CD138-positive cells on biopsy^‡^	80 (10–100)
Cytogenetics (FISH)	
1q gain^†^	21/47 (45)
13q deletion^†^	19/47 (40)
t(4;14)^†^	8/48 (17)
17p deletion^†^	2/41 (5)

ISS: international staging system; FISH: fluorescent in situ hybridization; *light chain type; ^†^absent values due to tests not done; the percentages are calculated based on the number of tests completed; ^‡^immunohistochemical stain on bone marrow biopsy.

**Table 2:** Comparison of clinical data in groups positive and negative for CD33 and CD13.

Clinical parameters	CD33 Mean or number (%)			CD13 Mean or number (%)		
	Negative (*N* = 44)	Positive (*N* = 10)	*P*	Negative (*N* = 18)	Positive (*N* = 20)	*P*
Age	61.2	61.1	0.978	61.9	60.4	0.688
Calcium (mg/dL)	9.03	9.78	0.049	9.03	9.60	0.145
Creatinine (mg/dL)	1.36	1.28	0.710	1.32	1.50	0.434
Albumin (mg/dL)	3.81	3.55	0.270	3.66	3.91	0.243
*β*2-Microglobulin (mg/dL)	4.76	4.71	0.966	3.09	4.28	0.635
Hemoglobin (g/dL)	10.6	9.8	0.277	10.3	10.6	0.687
LDH (U/L)	172	369	0.018	140	302	0.078
Monoclonal heavy chain			0.793			0.454
IgG	24 (77)	7 (23)		12 (60)	8 (40)	
IgA	10 (91)	1 (9)		2 (29)	5 (71)	
IgD	3 (100)	0 (0)		1 (33)	2 (67)	
Light chain only	7 (29)	2 (71)		3 (38)	5 (52)	
Monoclonal light chain			0.603			0.207
Kappa	27 (82)	6 (18)		9 (39)	14 (61)	
Lambda	17 (81)	4 (9)		9 (60)	6 (40)	
BM aspirate plasma cell (%)	38	48	0.903	36	48	0.198
Plasma cell type			0.022			0.519
Mature	35 (90)	4 (10)		14 (54)	12 (46)	
Immature	4 (50)	4 (50)		2 (29)	5 (71)	
Plasmablastic	1 (50)	1 (50)		0 (0)	1 (100)	
Pleomorphic	4 (80)	1 (20)		2 (50)	2 (50)	
Infiltration pattern			0.487			0.046
Interstitial	14 (88)	2 (12)		5 (100)	0 (0)	
Focal	2 (67)	1 (33)		1 (50)	1 (50)	
Diffuse	28 (80)	7 (20)		12 (44)	15 (56)	
Cytogenetics (FISH)^‡^						
t(4;14)	7/40	1/4	0.566	3/18	4/19	0.532
1q amplification	14/39	4/7	0.258	6/18	9/19	0.297
13q deletion	14/39	4/7	0.258	5/18	8/19	0.286
17p deletion	2/33	0/7	0.677	0/15	2/15	0.241
Cytogenetic high risk group^¶^	9/35	1/7	0.461	3/16	6/16	0.217
International staging system			0.742			0.647
Stage I	19 (86)	3 (14)		6 (43)	8 (57)	
Stage II	14 (78)	4 (22)		6 (43)	8 (57)	
Stage III	11 (79)	3 (21)		6 (60)	4 (40)	
Durie-Salmon stage			0.753			0.766
Stage I	5 (100)	0 (0)		2 (67)	1 (33)	
Stage II	9 (82)	2 (18)		3 (38)	5 (62)	
Stage III	30 (77)	9 (23)		13 (48)	14 (52)	
IMWG risk			0.867			0.791
Low	8 (82)	1 (18)		3 (60)	2 (40)	
Standard	30 (79)	8 (21)		12 (46)	14 (54)	
High	6 (86)	1 (14)		3 (43)	4 (47)	
IMWG response			0.742			0.698
Complete response	8 (80)	2 (20)		4 (50)	4 (50)	
Very good partial response	6 (86)	1 (14)		2 (40)	3 (60)	
Partial response	10 (83)	2 (17)		5 (71)	2 (29)	
Stable disease	1 (50)	1 (50)		1 (100)	0 (0)	
Progressive disease	5 (71)	2 (29)		3 (43)	4 (57)	

BM: bone marrow; LDH: lactate dehydrogenase; IMWG: International Myeloma Working Group; ^‡^numbers of positive cases among FISH tests done; percentages were not written because meanings were different from that of other parameters; ^¶^including t(4;14) or del(17p).

**Table 3:** Multivariate regression analysis of factors significantly associated with PFS and OS.

Variables	PFS			OS		
	HR	95% CI	*P*	HR	95% CI	*P*
CD13+	3.46	0.8–14.8	0.093	2.77	0.4–17.7	0.283
CD33+	3.86	1.1–13.7	0.037	13.8	3.1–61.3	0.001
*β*2-Microglobulin > 3.5 mg/dL	6.93	2.0–24.1	0.002	4.02	1.0–16.7	0.055
LDH > 202 U/L	1.84	0.5–6.9	0.370	2.88	0.6–14.2	0.195
Age ≥ 65 years	0.40	1.1–0.1	0.076	1.50	0.5–4.6	0.481
t(4;14)	0.51	0.1–2.2	0.368	1.21	0.2–6.4	0.823

PFS: progression free survival; OS: overall survival; HR: hazard ratio; CI: confidence interval.
